# Icariin and related metabolites in fibrosis management: pharmacological properties and molecular mechanism

**DOI:** 10.3389/fphar.2025.1619581

**Published:** 2025-06-04

**Authors:** Jiarui Zhao, Wei Zhang

**Affiliations:** College of First Clinical Medicine, Shandong University of Traditional Chinese Medicine, Jinan, Shandong, China

**Keywords:** icariin, icariside II, icaritin, fibrosis, pharmacokinetics, molecular mechanism

## Abstract

Fibrosis is a pathological hallmark of various chronic diseases and contributes significantly to organ dysfunction and poor clinical outcomes. Despite the availability of antifibrotic agents, their limited efficacy and adverse side effect profiles underscore the urgent need for safer and more effective therapeutic alternatives. Traditional Chinese medicines have emerged as promising candidates for fibrosis management. *Epimedium*, widely used in traditional Chinese medicine, exhibits notable antifibrotic activity, primarily attributed to its bioactive flavonoid icariin (ICA). However, the clinical application of ICA is hindered by its low bioavailability. Recent advances in extraction methods and drug delivery systems have improved the pharmacokinetic properties of ICA and related active metabolites, including icaritin and icariside II. These metabolites exert antifibrotic effects through multifaceted mechanisms, including anti-inflammatory and antioxidant activities, mitochondrial function modulation, apoptosis regulation, and autophagy. This review summarizes current insights into the molecular pathways through which ICA and related metabolites attenuate fibrosis, thereby supporting their potential for clinical translation in antifibrotic therapy.

## 1 Introduction

Fibrosis is caused by the abnormal accumulation of extracellular matrix (ECM), such as collagen, at the site of tissue injury. It can be induced by various stimuli, including infections and toxins (Wynn and Ramalingam, 2012; Wynn, 2008). Myofibroblasts and cytokines play important roles in the development of fibrosis (Henderson et al., 2020; Hinz and Lagares, 2020). Fibrosis occurs in a wide range of chronic diseases and is inextricably linked to immune, oxidative stress, and inflammatory responses (Antar et al., 2023). It can affect multiple organs, such as the lungs (Koudstaal et al., 2023), liver (Kisseleva and Brenner, 2021), kidneys (Huang et al., 2023), and heart (Gyöngyösi et al., 2017). Fibrotic organs often suffer irreversible functional damage, which can ultimately progress to organ failure. Consequently, the development of antifibrotic drugs has become a topic of significant interest. Some studies have identified molecules such as transforming growth factor-β (TGF-β) and LPA1 as promising targets for fibrosis treatment (Friedman et al., 2013). However, current antifibrotic therapies remain limited, underscoring the need to explore additional candidates with clinical potential.

Traditional Chinese medicine (TCM) has developed a unique and effective system of therapeutic theories and practices through centuries of clinical experience. Numerous studies have demonstrated the potential of TCM in treating liver fibrosis (Zhang and Schuppan, 2014), pulmonary fibrosis (Li and Kan, 2017), myocardial fibrosis (Ren et al., 2022), and other fibrotic conditions. Based on the 2020 edition of the Chinese Pharmacopoeia, *Epimedium* is the dried leaves of *Epimedium brevicornu* Maxim*., Epimedium sagittatum* (Sieb.et Zucc.) Maxim*., Epimedium pubescens* Maxim. or the *Epimedium koreanum* Nakai. *Epimedium*, also known as Xian Ling Pi, belongs to the Berberidaceae family and is widely used in clinical settings. According to TCM theory, *Epimedium* can “tonify kidney yang” and “strengthen muscles and bones” (Ma et al., 2011). It exhibits antioxidant and antiangiogenic properties (Xu et al., 2021) and has demonstrated therapeutic effects in a variety of conditions, including hepatic malignancies (Liu YM. et al., 2023), pulmonary malignancies (Xu et al., 2021), and osteoporosis (Indran et al., 2016; Xu et al., 2016). *Epimedium* contains various bioactive metabolites, with isoprenoid flavonoids being the primary metabolites (Ma et al., 2011). Among them, icariin (ICA) is one of the most prominent flavonoids. ICA is metabolized into several products, including icaritin, desmethyl icaritin, icariside I, and icariside II (Xu et al., 2007). Notably, icaritin and icariside II also exhibit antifibrotic properties.

Several studies have investigated the potential therapeutic effects of ICA and related metabolites in fibrotic diseases. This review aimed to summarize the pharmacological composition, pharmacokinetics, and antifibrotic mechanisms of ICA and related metabolites. We hypothesized that this study would support the application of ICA in the treatment of fibrosis and provide a reference for the development of more effective antifibrotic therapies in the future.

## 2 ICA and related metabolites

### 2.1 Pharmacokinetics of ICA and related metabolites

**FIGURE 1:**
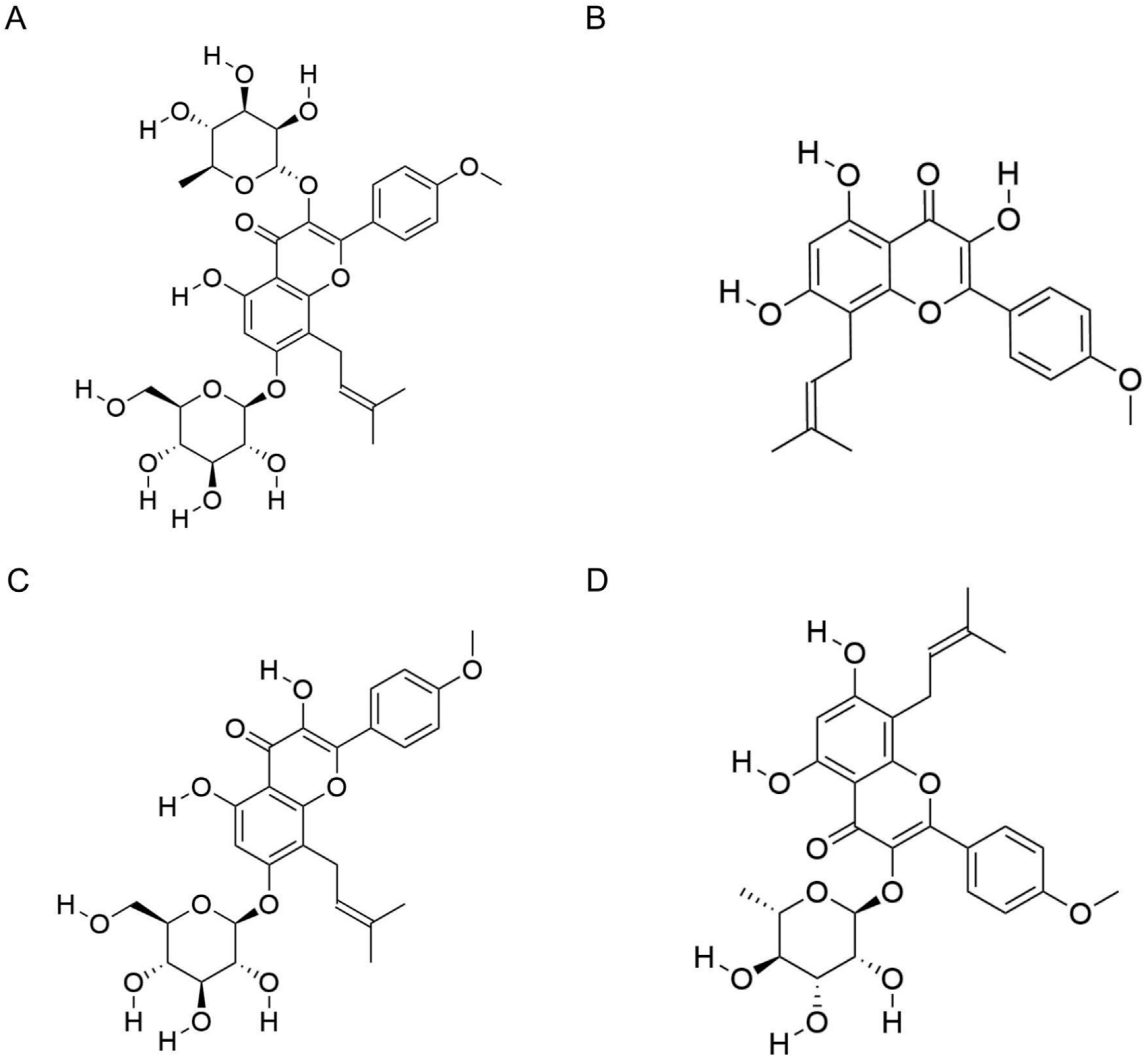
Molecular structures of icariin and related metabolites. **(A)** Molecular structure of icariin. **(B)** Molecular structure of icaritin. **(C)** Molecular structure of icariside I. **(D)** Molecular structure of icariside II.

Based on pharmacokinetic studies, we explored the absorption, metabolism, distribution, and excretion of ICA and related metabolites in biological systems. ICA (molecular formula: C_33_H_40_O_15_; molecular weight: 676.7 g/mol) is the principal active metabolite in *Epimedium*. The molecular structure of ICA is shown in Figure 1A. In the intestines, ICA is absorbed primarily in the form of icarisides I and II. However, ICA exhibits low oral bioavailability, and its half-life in rats is approximately 74 min (Cheng et al., 2016). The small intestine is the primary site of ICA metabolism, where both intestinal enzymes and microbiota play important roles (Zhou et al., 2013).

Following phase I metabolism—such as demethylation and deglycosylation—ICA is converted into various metabolites, including icaritin, icariside I, and desmethyl icaritin. During phase II metabolism, ICA undergoes conjugation to form glucuronide metabolites such as icaritin-7-O-glucuronide (icaritin-7-O-gluA) and icaritin-3-O-rhamnoside-7-O-glucuronide (icaritin-3-O-rha-7-O-gluA) (Qian et al., 2012). ICA has limited ability to cross the blood–brain barrier and is rarely distributed in brain tissue. The excretory metabolites of ICA vary with the route of administration; ten metabolites have been identified in the feces of rats after oral administration, compared to nine following intramuscular administration.

ICA is metabolized into several active derivatives, including icaritin (molecular formula: C_21_H_20_O_6_; molecular weight: 368.4 g/mol), and icariside I (molecular formula: C_27_H_30_O_11_, molecular weight: 530.5 g/mol) and icariside II (molecular formula: C_27_H_30_O_10_, molecular weight: 514.5 g/mol). The molecular structures are shown in Figures 1B–D. After entering the bloodstream, these metabolites are distributed to various tissues and organs *via* systemic circulation. Plasma metabolite profiling has identified 19 metabolites, including icaritin, icariside II, and icariside-3-O-gluA (Zhou et al., 2013).

Icaritin exhibits high lipophilicity and low water solubility, and its distribution and excretion profiles vary with the route of administration. The half-life of orally administered icaritin (λz: 8.3 ± 1.0 h) is longer than that of ICA, with most of the drug accumulating in the liver and being excreted *via* urine (Zhang B. et al., 2017). Similarly, intraperitoneally administered icaritin also exhibits a longer half-life than ICA (λz: 3.14 ± 0.34 h), with predominant distribution in the kidneys and liver (Huang et al., 2019).

Icariside II is a major metabolite found in plasma following oral administration of ICA in rats, and its conversion is significantly influenced by the intestinal microenvironment. Approximately 91.2% of orally administered ICA is transformed into icariside II in the intestine, whereas only 0.4% of intravenously administered ICA undergoes this conversion (Cheng et al., 2015).

### 2.2 Delivery of ICA and related metabolites

The direct uptake of ICA and related metabolites *in vivo* is limited, highlighting the clinical value of developing more efficient drug modifications and delivery strategies. Alginate and chitosan, two natural polysaccharides widely used in biomedicine, have been employed to encapsulate ICA into microspheres. This formulation protects ICA from gastric degradation and enables targeted intestinal release, allowing ICA to remain in the colon for over 12 h (Wang et al., 2016).

With the advancement of nanotechnology, numerous studies have explored its application in drug delivery. Compared to traditional methods, nanotechnology offers several advantages, including enhanced drug stability, prolonged systemic activity, targeted tissue penetration, and improved delivery efficiency (Lu et al., 2023). Various types of ICA and their metabolite-based formulations have been developed. Liposomes and micelles are commonly used carriers for ICA and related metabolites. Some studies have also designed ICA-loaded nanogels. Notably, sustained release of ICA *via* nasal mucosal delivery has been employed in antidepressant therapy targeting the central nervous system (Xu et al., 2020).

Icariside II has been combined with phospholipids, taking advantage of the high intestinal permeability of phospholipids to enhance systemic absorption (Jin et al., 2012). Furthermore, incorporating icariside II into Solutol HS 15-modified liposomal lecithin has enabled sustained drug retention in pulmonary tumor tissues, supporting the advancement of precision medicine strategies for ICA metabolites (Yan et al., 2016).

pH-sensitive micellization techniques have been used to encapsulate icaritin in the hydrophobic core of polymeric micelles, thereby protecting it from enzymatic degradation and significantly enhancing oral bioavailability under simulated physiological conditions (Tang et al., 2021). Additionally, co-delivery systems integrating icaritin and doxorubicin *via* polylactic-co-glycolic acid-polyethylene glycol-aminoethyl anisamide nanoparticles (NPs) have shown improved circulation time and pharmacokinetics, prolonging the half-life of free icaritin (Yu et al., 2020).

Current research on drug delivery systems reveals promising potential. NP-based technologies and other innovative approaches may substantially enhance the therapeutic application and clinical efficacy of ICA and related metabolites. Our review of these delivery strategies provides valuable insights into expanding their therapeutic utility (Table 1).

**TABLE 1:** Delivery methods of icariin and related metabolites.

Deliveries	Delivery Type	Advantage	Research Levels	References
Icariin	The alginate-chitosan microspheres	Reduction of gastric fluid depletion of icariin	*in vitro* and *in vivo*	Wang et al. (2016)
Icariin	A nanogel-thermoresponsive hydrogel compound system	Antidepressant treatment in the central nervous system	*in vitro* and *in vivo*	Xu et al. (2020)
Icariside II	The mixed micelles based on lecithin and Solutol HS 15	Tumor-targeted delivery of icariside II	*in vitro* and *in vivo*	Yan et al. (2016)
Icaritin	The mixed micelles of Soluplus and Poloxamer 407	Increased utilization of oral icaritin	*in vitro* and *in vivo*	Tang et al. (2021)
Icaritin and Doxorubicin	The poly lactic-co-glycolic acid-polyethylene glycol-aminoethyl anisamide nanoparticle targeted codelivery	Reduced icaritin clearance in the circulation	*in vitro* and *in vivo*	Yu et al. (2020)

### 2.3 Extraction of ICA and related metabolites

The limited bioavailability of natural *Epimedium* derivatives in humans underscores the necessity of employing advanced techniques to obtain high-purity isolates. Traditional extraction methods, such as thermal reflux and ethanol-based techniques (Zhou et al., 2020), have been partially adopted in industrial applications. However, recent innovations have significantly improved extraction efficiency. Details are presented in Table 2. Ultrasound-assisted extraction (Zhang et al., 2008; Guo et al., 2020), chromatographic techniques (Liu et al., 2005), and microwave-mediated processes (Huang et al., 2005) offer superior yields and processing speeds. Notably, the integration of deep eutectic solvents with ultrasound-assisted extraction has reduced ICA processing time by 25% while maintaining yield (Guo et al., 2020). High-speed countercurrent chromatography, a carrier-free liquid-liquid partitioning system, enables high-purity isolation of ICA (99.7%), icariside I (98.2%), and icariside II (98.5%), owing to optimized phase dynamics and solvent systems (Liu et al., 2005). Microwave-assisted extraction further improves efficiency, halving the isolation time and increasing ICA yield by 7% through dielectric heating and enhanced cell wall disruption (Huang et al., 2005). These advanced approaches offer substantial benefits, including higher separation efficiency and reduced raw material consumption.

**TABLE 2:** Extraction methods of icariin and related metabolites.

Extracted metabolites	Extraction Technology	Advantage	References
Icariin, Epimedin A, Epimedin B, Epimedin C	Ultrasonic-assisted extraction	Short extraction time	Zhang et al. (2008)
Icariin, Icariside II, Epimedin A, Epimedin B, Epimedin C	Deep eutectic solvent combined with ultrasound-assisted extraction technology	Low solvent consumption, short extraction time	Guo et al. (2020)
Icariin, Icariside II, Epimedokoreanoside I	High-speed counter-current chromatography technology	High separation purity	Liu et al. (2005)
Icariin	Microwave technology	High separation purity	Huang et al. (2005)
Icariin, Icariside II	GH78 α-L-rhamnosidase AmRha extraction technology	High extraction rate	Zhang et al. (2023)
Icaritin	Whole-cell catalysis	High extraction efficiency, simplified production step	Lin et al. (2023)
Icaritin, Genistein	Flavonoid glycosides/β-cyclodextrin inclusion complex extraction technology	High substrate solubility, short extraction time	Jin et al. (2013)
Icariside II	4LP-Tpebgl3@Na-Y immobilized enzyme extraction technology	High enzyme stability and utilization	Lu et al. (2022)

Enzymolysis has emerged as a sustainable and efficient strategy for ICA and metabolite extraction, with advantages such as environmental compatibility, mild operating conditions, and process simplification. Key enzymes involved in the biotransformation cascade include α-L-rhamnosidases, β-glucosidases, and xylanases. α-L-rhamnosidases catalyze the hydrolysis of Epimedin C to ICA, which is subsequently converted to icariside I *via* rhamnosyl residue cleavage (Zhang et al., 2023; Cheng et al., 2022). Notably, α-L-rhamnosidase (AmRha) derived from *Aspergillus mulundensis* achieved a 92.3% ICA conversion rate under optimized conditions (Zhang et al., 2023). β-Glucosidases facilitate the deglycosylation of ICA intermediates to generate bioactive icaritin, with engineered systems enabling high selectivity and yield (Lin et al., 2023; Xie et al., 2020). A major advancement is the development of whole-cell catalytic systems co-expressing α-L-rhamnosidase SPRHA2 and β-glucosidase PBGL, achieving a 95.23% icaritin yield in a single-step bioreaction (Lin et al., 2023). In addition, β-xylosyl hydrolase can convert Epimedin B to ICA (Su et al., 2023). To reduce reaction time and enhance substrate availability, recent studies have employed β-cyclodextrin to improve ICA solubility *via* host-guest complexation (Jin et al., 2013). Enzyme immobilization strategies have also been employed to enhance catalytic efficiency and operational stability. For instance, the covalent binding of β-glucosidase to 4LP carriers yielded the 4LP-Tpebgl3@Na-Y enzyme, which demonstrated superior activity and reusability compared to free enzymes (Lu et al., 2022).

## 3 Mechanisms of ICA and related metabolites

**FIGURE 2:**
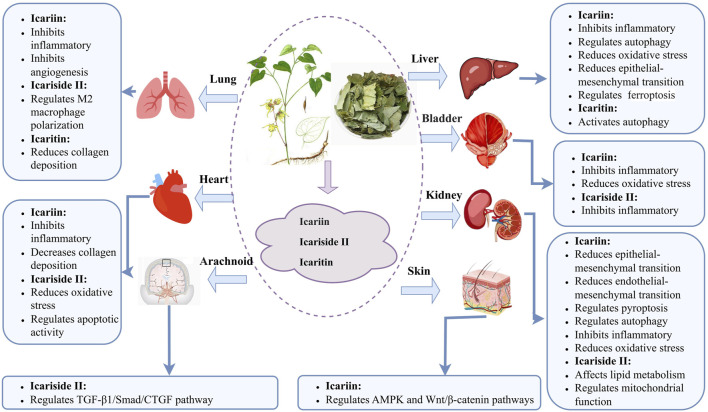
Antifibrotic mechanisms of icariin and related metabolites in different organs.

Aberrant inflammatory activity and oxidative stress are hallmark features of fibrosis initiation and progression. ICA and related metabolites exert antifibrotic effects by downregulating inflammatory cytokines and attenuating oxidative stress. These metabolites reduce collagen deposition and ameliorate fibrotic pathology *via* multiple mechanisms, including modulation of macrophage polarization, autophagy, apoptosis, and pyroptosis (Figure 2). Cell and animal studies have shown several signaling pathways in the antifibrotic actions of ICA and related metabolites, particularly TGF-β1/Smad, nuclear factor kappa B (NF-κB), AMP-activated protein kinase (AMPK), Nrf-2/HO-1, and WNT/β-catenin. Moreover, ICA and related metabolites regulate microRNA expression, which in turn modulates downstream gene targets and contributes to their therapeutic potential. We have summarized the antifibrotic effects and mechanisms for ICA and related metabolites in Table 3. The level of the included research has also been clarified in Table 3.

**TABLE 3:** Mechanisms of icariin and related metabolites.

Organ	Metabolite	Mechanisms	Research Levels	References
Lungs	Icariin	Inhibits Hippo/YAP pathway and Endocannabinoid signalling/cannabinoid receptor 2 pathway. Reduces inflammation and collagen deposition. Inhibits the mTOR and the P70S6 kinase expression. Regulates angiogenesis and autophagy phenomena	Omics research. Cell research. Animal research	Du et al. (2021), Du et al. (2022), Wang et al. (2021a)
	Icariside II	Downregulates WNT/β-catenin pathway and PI3K/Akt/β-catenin pathway. Attenuates M2 macrophage polarization	Cell research. Animal research	Deng et al. (2023), Deng et al. (2024)
	Icaritin	Increases PPARγ expression. Decreases collagen deposition	Cell research. Animal research	Hua et al. (2021)
Heart	Icariin	Inhibits the expression of c-Jun and p65. Targets TGF-β1/Smad pathway. Decreases collagen expression	Omics research. Cell research. Animal research	Zhang et al. (2021), Jia et al. (2023), Qiao et al. (2020)
	Icariside II	Targets ASK1-JNK/p38 pathway. Inhibits apoptosis. Activates AMPK pathway. Inhibits mTORC1/p70S6k pathway, NF-kB pathway, TGF-β1/Smad2 pathway, MMP/TIMP-1 pathway and TGF-β/Smad2,3/p-p38 pathway. Decreases collagen aggregation	Cell research. Animal research	Wu et al. (2018), Fu et al. (2018), Fu et al. (2020), Liu et al. (2018)
Kidneys	Icariin	Downregulates the miR-320a-3p expression. Restores the BMP6 expression. Modulates Smad pathway, IL-1β/TGF-β pathway, NF-κB pathway, NF-κB/NLRP3 pathway, AMPK/SIRT1/NF-κB pathway, AMPK/ACC pathway, TLR4/NF-κB pathway. Reduces inflammatory infiltration and oxidative stress. Inhibits Notch2/Hes-1 pathway. Inhibits epithelial-mesenchymal transition. Targets AR/RKIP/MEK/ERK pathway. Inhibits endothelial-mesenchymal transition. Inhibits the NLRP3 inflammatory vesicles. Inhibits pyroptosis. Activates Nrf2/HO-1 pathway and GPER expression. Improves mitochondrial function. Regulates miR-192-5p/GLP-1R pathway. Restores autophagy function	Cell research. Animal research	Wang et al. (2021b), Qi et al. (2021), Chen et al. (2019), Hou et al. (2022), Zhang et al. (2022), Yao et al. (2024), Wang et al. (2024a), Zhao et al. (2023), Zhang et al. (2017b), Duan et al. (2024), Ding et al. (2024), Xie et al. (2022), Jia et al. (2021)
	Icariside II	Restores PPARα-mediated fatty acid oxidation. Enhances mitochondrial antioxidant capacity and energy metabolism	Cell research. Animal research	Wang et al. (2024b)
Liver	Icariin	Upregulates the expression of miR-875-5p. Inhibits Hedgehog pathway. Inhibits epithelial-mesenchymal transition. Activates Nrf2-xCT/GPX4 pathway. Inhibits ferroptotic. Inhibits autophagy and angiogenesis	Cell research. Animal research	Ye et al. (2020), Choi et al. (2023), Algandaby et al. (2017)
	Icaritin	Upregulates Bak-1, Bmf, and Bax. Regulates apoptosis	Omics research. Cell research. Animal research	Li et al. (2011)
Bladder	Icariin	Regulates Nrf-2/HO-1 pathway and NF-κB pathway. Reduces inflammatory response and oxidative stress	Cell research. Animal research	Amanat et al. (2022)
	Icariside II	Regulates the expression of H3F3C, ISG15, SPP1 and LCN2. Reduces the inflammatory response	Omics research. Cell research. Animal research	Sun et al. (2024)
Skin	Icaritin	Activates AMPK pathway. Inhibits Wnt/β-catenin pathway	Cell research	Li et al. (2021)
Arachnoid	Icariside II	Regulates TGF-β1/Smad/CTGF signaling pathway	Animal research	Dong et al. (2018)

### 3.1 Lungs

Fibrosing interstitial lung diseases (F-ILDs) represent a heterogeneous group of chronic, progressive pulmonary disorders characterized by aberrant fibroblast activation and excessive ECM deposition. The prognosis of F-ILD remains poor, with over 50% of patients succumbing to respiratory failure or related complications within 5 years of diagnosis (Kamiya et al., 2024). Although the etiology of some F-ILD subtypes is idiopathic, others are attributable to autoimmune diseases, drug-induced toxicity, environmental allergens, or occupational exposures (Parimon et al., 2021). Clinically, the F-ILD spectrum includes idiopathic pulmonary fibrosis, hypersensitivity pneumonitis, and connective tissue disease-associated interstitial lung disease.

Chronic inflammation stimulation plays a central pathogenic role in F-ILD by promoting aberrant fibroblast activation, sustained secretion of proinflammatory mediators, and excessive collagen deposition. ICA has demonstrated therapeutic potential in ameliorating pulmonary inflammation and fibrotic progression. ICA downregulates the expression of key inflammatory cytokines, including tumor necrosis factor-α (TNF-α) and interleukin-1β (IL-1β), and modulates immune cell distribution, particularly leukocytes and neutrophils. Through regulation of the Hippo/Yes-associated protein signaling pathway, ICA inhibits the expression of α-smooth muscle actin (α-SMA) and TGF-β, thereby attenuating collagen deposition and fibroblast activation (Du et al., 2021).

Sustained inflammatory signaling in F-ILD frequently promotes pathological angiogenesis, which further exacerbates fibrotic remodeling. Metabolomic profiling of patients with IPF has identified alterations in metabolites linked to the endocannabinoid pathway, which is active in pulmonary macrophages and exerts anti-inflammatory and antiangiogenic effects. ICA exerts antifibrotic actions by modulating the endocannabinoid/cannabinoid receptor two axis, thereby suppressing pathological angiogenesis and inflammation (Du et al., 2022). To overcome the rapid systemic clearance of ICA and enhance pulmonary targeting, lipid-based NP formulations have been developed. These nanocarriers demonstrated improved bioavailability and therapeutic efficacy in experimental models by suppressing pathological angiogenesis in fibrotic lung tissues (Wang J. et al., 2021).

Icariside II, a major metabolite of ICA, has shown robust antifibrotic effects in both preclinical and emerging clinical studies of pulmonary fibrosis. Macrophages play a pivotal role in fibrotic lung disease by regulating inflammatory responses and tissue remodeling. They polarize into classically activated M1 characterized by CD86 and inducible nitric oxide synthase expression – (and alternatively activated M2 macrophages) – marked by CD163 and CD206 expression. Although M1 macrophages are proinflammatory, M2 macrophages are associated with tissue repair and fibrosis, often induced by cytokines such as IL-3, interleukin-4 (IL-4), toll-like receptor (TLR) ligands, immune complexes, or A2 adenosine receptor agonists (Liu et al., 2014). Notably, M2-like interstitial macrophages have been implicated in the initiation and progression of pulmonary fibrosis (Wynn and Vannella, 2016).

Icariside II downregulates the expression of M2 macrophage markers, including CD163, arginase-1, and CD206, effectively reducing M2 macrophage accumulation in fibrotic lungs. This antifibrotic activity is mediated through inhibition of the WNT/β-catenin and PI3K/Akt signaling pathways, which are central to macrophage polarization and fibroblast activation. By attenuating M2 polarization, icariside II mitigates immune dysregulation and limits pulmonary tissue injury in fibrotic models (Deng et al., 2023; Deng et al., 2024).

Furthermore, icaritin, another bioactive metabolite of ICA, exhibits both prophylactic and therapeutic efficacy in murine models of pulmonary fibrosis. Icaritin upregulates the expression of peroxisome proliferator-activated receptor gamma (PPARγ), a key nuclear receptor involved in antifibrotic signaling. Activation of PPARγ reduces collagen deposition and decelerates fibrotic progression in the lung parenchyma (Hua et al., 2021).

### 3.2 Heart

Myocardial fibrosis, a hallmark of pathological cardiac remodeling, underlies the progression of diverse cardiovascular disorders. It is commonly observed in the advanced stages of diabetic cardiomyopathy (DCM), hypertension, myocardial infarction, Takotsubo syndrome, and other related conditions (Zhang et al., 2021; Jia et al., 2023; Qiao et al., 2020; Qi et al., 2019; Wu et al., 2018; Fu et al., 2018; Fu et al., 2020). Clinically, myocardial fibrosis contributes to maladaptive myocardial hypertrophy and eventual heart failure, marking the transition from compensatory adaptation to decompensated dysfunction. It is a major determinant of ventricular stiffening, impaired contractility, and poor cardiovascular outcomes, thereby significantly increasing morbidity and mortality.

Functional impairment of the myocardium is an early feature of fibrotic cardiac remodeling. In preclinical models of DCM, cardiac dysfunction has been linked to decreased expression of endothelial nitric oxide synthase 3 (NOS3) and increased levels of phosphodiesterase 5A (PDE5A) within myocardial tissues. ICA has been shown to modulate the expression of both NOS3 and PDE5A in DCM models. By targeting the PDE5/NOS3 axis and the downstream sGC-cGMP-PKG signaling pathway, ICA alleviates cardiac dysfunction and attenuates myocardial fibrosis in DCM rats (Zhang et al., 2021). Inflammatory cell infiltration and ECM deposition are key processes in the progression of myocardial fibrosis. Analogous to F-ILD, cardiac fibrotic tissues demonstrate elevated expression of proinflammatory cytokines such as interleukin-13 and TGF-β1. Excessive collagen accumulation disrupts myofibril alignment and compromises structural integrity. ICA suppresses collagen deposition and mitigates myocardial fibrosis in DCM by downregulating the expression of p65 and JUN, two pivotal transcription factors involved in fibrotic signaling (Zhang et al., 2021). In addition, ICA reduces fibronectin and collagen expression while inhibiting inflammatory cytokine infiltration. Through modulation of the TGF-β1/Smad pathway, ICA significantly attenuates myocardial fibrosis progression in both myocardial infarction and DCM (Jia et al., 2023; Qiao et al., 2020), underscoring its antifibrotic efficacy and cardioprotective potential.

Icariside II, a bioactive metabolite of ICA, has also demonstrated potent antifibrotic effects in the heart. Oxidative stress plays a central role in myocardial fibrogenesis, with excessive reactive oxygen species (ROS) contributing to aberrant apoptosis and fibroblast activation. Icariside II suppresses the expression of ASK1, JNK, and p38, thereby mitigating oxidative stress in hypertensive cardiomyocytes. As a tightly regulated mechanism of programmed cell death, apoptosis maintains tissue homeostasis and exhibits dual regulatory effects in fibrotic progression as a programmed cell death mechanism characterized by strict regulation and orderly execution. Dysregulated apoptotic activity has been implicated in excessive accumulation of inflammatory factors and myocardial fibrotic remodeling. Icariside II exerts antifibrotic effects by modulating the ASK1-JNK/p38 axis, downregulating pro-apoptotic proteins such as Bax and p53, and reducing collagen deposition and fibroblast overactivation. These actions demonstrate its capacity to correct apoptosis-related dysregulation at the cellular and pathway-specific levels (Wu et al., 2018).

Further mechanistic studies have revealed that icariside II alleviates myocardial fibrosis in hypertension by inhibiting the TGF-β1/Smad2, NF-κB/p65, and MMP/TIMP-1 signaling pathways (Fu et al., 2018; Fu et al., 2020). Moreover, it suppresses fibrotic cardiac remodeling in models of cardiac hypertrophy through regulation of the mTORC1/p70S6k pathway, highlighting its multi-targeted therapeutic potential in the management of myocardial fibrosis (Liu et al., 2018).

### 3.3 Kidneys

Chronic kidney disease (CKD) encompasses a broad range of pathological conditions, including primary and secondary glomerulonephritis, tubular injury, and renal vascular disorders. Renal fibrosis represents the common pathological endpoint of these conditions and is a principal driver of progressive functional deterioration leading to end-stage renal disease. Key mechanisms underpinning renal fibrogenesis include pyroptosis, dysregulated autophagy, and mitochondrial dysfunction. Despite the clinical burden of renal fibrosis, effective antifibrotic therapies remain scarce. Accumulating evidence suggests that ICA and related active metabolites exert renoprotective effects by attenuating renal fibrosis and preserving kidney function.

ICA improves renal function in CKD models by reducing 24-h urinary protein excretion, serum creatinine, and blood urea nitrogen levels (Wang M. et al., 2021; Qi et al., 2021). It also alleviates renal injury and fibrosis by limiting collagen deposition (Chen et al., 2019; Hou et al., 2022). ICA exerts its antifibrotic effects through multiple molecular pathways: it inhibits epithelial–mesenchymal transition (EMT) *via* the Notch2/Hes-1 pathway (Zhang et al., 2022); suppresses endothelial–mesenchymal transition in diabetic nephropathy by modulating the AR/RKIP/MEK/ERK cascade (Yao et al., 2024); and mitigates fibrosis by downregulating miR-320a-3p, thereby restoring BMP6 expression (Wang K. et al., 2024).

Consistent with its effects in pulmonary and cardiac fibrosis, ICA attenuates inflammatory activation and oxidative stress-two primary drivers of renal fibrogenesis. It suppresses proinflammatory cytokines and immune cell infiltration by regulating the TLR4/NF-κB, NF-κB/NLRP3, AMPK/SIRT1/NF-κB, AMPK/ACC, and IL-1β/TGF-β pathways. Simultaneously, ICA enhances antioxidant defense by increasing superoxide dismutase (SOD) and catalase (CAT) activities, as well as serum total antioxidant capacity, thereby slowing fibrotic progression in CKD (Wang M. et al., 2021; Qi et al., 2021; Chen et al., 2019; Hou et al., 2022; Zhao et al., 2023; Zhang L. et al., 2017).

Pyroptosis, a form of inflammatory necrosis, is a distinct programmed cell death mechanism implicated in renal pathophysiology. When activated during pyroptosis, gasdermin proteins embed into the cell membrane to form pores, triggering the release of cellular contents and amplifying inflammatory cascades (Elias et al., 2023). Given its capacity to release abundant proinflammatory mediators, pyroptosis plays a critical role in promoting renal fibrogenesis (Liu Y. et al., 2023). A previous study demonstrated that ICA downregulated the expression of NLRP3, GSDMD, caspase-1, ASC, and Ly6C in the renal tissues of patients with nephrotic syndrome, concurrently reducing the proportion of TUNEL-positive cells. By suppressing NLRP3 inflammasome activation, ICA inhibits pyroptosis-driven inflammation and attenuates renal fibrosis (Duan et al., 2024). Mitochondria are the primary sites of intracellular ROS generation and mediate oxidative stress and secondary inflammatory responses. In fibrotic kidneys, mitochondrial dysfunction manifests as structural abnormalities–including swelling, cristae disruption, and membrane potential depolarization–which exacerbate ROS accumulation and pathological cytokine secretion. ICA has been shown to upregulate the Nrf2/HO-1 pathway, thereby mitigating oxidative stress and inflammation in CKD. This modulation restores the mitochondrial morphology and bioenergetic function, significantly ameliorating tubulointerstitial fibrosis (Ding et al., 2024). Emerging evidence indicates that ICA enhances the expression of G protein-coupled estrogen receptors and upregulates the mitochondrial biogenesis regulators, including peroxisome proliferator-activated receptor gamma coactivator 1-alpha and carnitine palmitoyltransferase 1-alpha. ICA thereby ameliorates mitochondrial dysfunction and attenuates renal fibrosis (Xie et al., 2022).

The interaction between autophagy and renal fibrosis is complex. Although autophagy serves as a homeostatic mechanism to maintain cellular equilibrium, it exhibits dual, context-dependent roles–either suppressing or promoting fibrotic progression under distinct pathological conditions (Ruby et al., 2023). For instance, inhibition of the mTORC1 pathway activates autophagy but paradoxically exacerbates tubular injury and renal fibrosis (Shi et al., 2025). Conversely, the overexpression of hematopoietic cell kinases suppresses autophagic flux, thereby aggravating renal inflammation and fibrotic remodeling (Chen et al., 2023). In diabetic nephropathy, ICA suppresses miR-192-5p overexpression while elevating the expression of glucagon-like peptide-1 receptor and microtubule-associated protein one light chain 3-II, a marker of autophagosome formation. Through autophagy induction, ICA downregulates fibrotic markers such as alpha smooth muscle actin and collagen I, highlighting its therapeutic potential in mitigating renal fibrosis (Jia et al., 2021).

Icariside II, a bioactive metabolite of ICA, has shown therapeutic potential against renal fibrosis. Dysregulated lipid metabolism is implicated in the pathogenesis of renal fibrosis, with lipotoxicity and fatty acid metabolic dysfunction serving as central mechanisms driving interstitial fibrogenesis. Experimental studies have revealed that icariside II not only attenuates renal fibrosis in CKD rat models but also preserves mitochondrial function by reducing serum-free fatty acid levels and renal triglyceride accumulation. Mechanistically, icariside II modulates lipid metabolism-associated pathways and gene expression in fibrotic kidneys. Through upregulation of PPARα expression, icariside II enhances the expression of fatty acid oxidation-related proteins such as CPT-1α and ACADSB, mitigates lipid deposition, and improves mitochondrial antioxidant capacity and NAD+/NADH ratios. These coordinated actions effectively suppress renal interstitial fibrosis during CKD progression (Wang M. et al., 2024).

### 3.4 Liver

Hepatic fibrosis, characterized by excessive ECM deposition, constitutes a pivotal step in the progression of chronic liver diseases—including non-alcoholic fatty liver disease and viral hepatitis—to cirrhosis. With a high global incidence and mortality, hepatic fibrosis is strongly associated with metabolic comorbidities such as obesity, diabetes mellitus, and hypertension (Man et al., 2023). Hepatic stellate cells (HSCs), the predominant precursors of liver myofibroblasts, undergo pathological activation, which drives ECM overproduction and fibrotic remodeling.

ICA inhibits EMT and attenuates hepatic fibrosis by downregulating fibrotic and mesenchymal markers while upregulating epithelial markers in primary HSCs. In hepatic fibrosis, miR-875-5p is markedly suppressed, and GLI1 has been identified as a direct downstream effector. ICA upregulates miR-875-5p and concurrently downregulates GLI1 and Hedgehog signaling, thereby inhibiting HSC activation (Ye et al., 2020).

Emerging evidence suggests that ferroptosis—a regulated form of cell death driven by lipid peroxidation, antioxidant dysfunction, and iron dysregulation—contributes to hepatic fibrosis. Glutathione peroxidase 4 (GPX4) plays a central role by neutralizing lipid peroxides and maintaining redox equilibrium (Jiang et al., 2021). ICA ameliorates hepatic fibrosis and steatosis in methionine–choline-deficient diet-fed mice by downregulating ferroptosis-related proteins, including ACSL4, AIF, and ALOX12. ICA activates the Nrf2-xCT/GPX4 axis, thereby reducing iron-dependent lipid peroxidation and mitigating ferroptotic injury (Choi et al., 2023). Concurrently, ICA reduces malondialdehyde (MDA) accumulation and inflammatory cell infiltration while enhancing SOD activity. These multimodal effects highlight ICA’s anti-autophagic, antiangiogenic, anti-inflammatory, anti-ferroptotic, and antioxidant capacities in hepatic fibrosis (Algandaby et al., 2017).

Icaritin, a major metabolite of ICA, also exerts significant hepatoprotective effects. It reduces serum levels of aspartate aminotransferase and alanine aminotransferase, suppresses HSC activation, and limits collagen deposition in fibrotic liver tissue. Mechanistically, icaritin promotes mitochondrial apoptosis of activated HSCs by upregulating pro-apoptotic proteins (Bak-1, Bmf, and Bax) and downregulating the anti-apoptotic protein Bcl-2. This pro-apoptotic shift contributes to the resolution of fibrogenesis and restoration of hepatic function (Li et al., 2011).

### 3.5 Bladder

Bladder fibrosis is a pathological hallmark of chemotherapy- and radiotherapy-induced cystitis. Cyclophosphamide (CYP), a widely utilized chemotherapeutic agent, induces cystitis through direct urothelial toxicity and inflammatory activation. ICA mitigates CYP-induced cystitis and associated fibrotic changes by downregulating proinflammatory cytokines (IL-6, TNF-α, and IL-1β) and upregulating anti-inflammatory mediators (IL-10 and IL-4). Additionally, ICA enhances antioxidant enzyme activity—including glutathione, glutathione S-transferase, SOD, and CAT—while reducing MDA and myeloperoxidase levels, thereby suppressing oxidative stress. These effects are mediated through coordinated regulation of the Nrf-2/HO-1 and NF-κB pathways, culminating in reduced inflammatory infiltration and fibrotic remodeling (Amanat et al., 2022).

Icariside II has also demonstrated efficacy in ameliorating radiation-induced bladder fibrosis. Radiotherapy is a significant contributor to post-treatment cystitis, which manifests clinically as pelvic pain, hematuria, and progressive fibrotic bladder dysfunction. In preclinical models, icariside II reduces inflammatory mediators and collagen deposition, enhances bladder compliance, and increases functional capacity. Transcriptomic sequencing and molecular docking studies have identified high-affinity interactions between icariside II and molecular targets such as H3F3C, ISG15, SPP1, and LCN2, implicating these genes in its therapeutic mechanism (Sun et al., 2024). These findings highlight the potential of icariside II as a targeted therapeutic agent for radiation-induced bladder fibrosis.

### 3.6 Skin

Scleroderma is a connective tissue disorder characterized by progressive skin thickening and fibrosis, with a higher incidence among females. The disease exists in two primary forms: localized scleroderma, which predominantly involves cutaneous fibrosis and is typically associated with a favorable prognosis; and systemic scleroderma, which affects multiple organs, involves elevated circulating autoantibodies, and carries a poorer clinical outcome. ICA has demonstrated antifibrotic efficacy in experimental models of dermal fibrosis. Mechanistically, ICA activates AMPK signaling while concurrently inhibiting the WNT/β-catenin pathway. This dual regulatory effect leads to the downregulation of collagen genes (COL1A1, COL1A2, and COL3A1) and key fibrotic markers, including connective tissue growth factor (CTGF) and α-SMA, thereby attenuating dermal fibrotic remodeling (Li et al., 2021).

### 3.7 Arachnoid

Chronic hydrocephalus is a frequent complication following subarachnoid hemorrhage and is primarily driven by subarachnoid fibrosis, which impairs cerebrospinal fluid (CSF) circulation. Icariside II has been shown to ameliorate hydrocephalus and associated neurocognitive dysfunction by targeting the fibrogenic TGF-β1/Smad/CTGF signaling axis. Specifically, icariside II suppresses the expression of TGF-β1, phosphorylated Smad2/3, and CTGF, thereby reducing fibrotic remodeling within the subarachnoid space. This intervention mitigates pathological ventricular dilation and preserves CSF dynamics, underscoring the therapeutic potential of icariside II in managing post-hemorrhagic hydrocephalus (Dong et al., 2018).

## 4 Discussion

Antifibrotic drugs are currently limited. Pirfenidone and nintedanib are common antifibrotic agents. Nintedanib reduces pulmonary fibrosis by inhibiting the tyrosine kinase pathway (Flaherty et al., 2019). Pirfenidone reduces pulmonary fibrosis through anti-inflammatory effects (Torre et al., 2024). Both pirfenidone and nintedanib have a higher frequency of side effects (Richeldi et al., 2014; Taniguchi et al., 2010). *Epimedium*, a traditional Chinese medicinal botanical drug, has demonstrated considerable therapeutic promise in the management of fibrotic disorders. *Epimedium* is often involved in the treatment of fibrosis, either alone or in a formula. A randomized controlled clinical study was conducted comparing a formula containing *E*pimedium with pirfenidone. The results showed that compared to the pirfenidone group, the formula containing *Epimedium* group had a lower incidence of adverse effects (Cao, 2023). ICA is the main bioactive metabolite of *Epimedium*. A large number of cell and animal studies have confirmed that ICA and related metabolites have good antifibrotic effects. Regrettably, there are some gaps in the clinical controlled studies of ICA and related metabolites with other antifibrotic drugs. In the future, it is necessary to carry out more large-scale clinical controlled experiments on ICA and related metabolites, so as to better compare and contrast ICA with other antifibrotic drugs in a side-by-side discussion.

Advances in drug delivery technologies have markedly improved the bioavailability and systemic distribution of ICA and related metabolites. These metabolites exhibit potent anti-inflammatory and antioxidant properties and exert broad-spectrum antifibrotic effects by modulating key pathological processes, including EMT, macrophage polarization, autophagy, apoptosis, pyroptosis, and ferroptosis. Through these mechanisms, ICA and related metabolites attenuate collagen deposition and inhibit aberrant fibroblast activation, thereby exerting therapeutic effects across diverse organ systems, including the lungs, heart, kidney, liver, bladder, skin, and subarachnoid space. This review found some contradiction in the current basic studies related to ICA and related metabolites. In renal fibrosis, ICA can restore autophagy by modulating the miR-192-5p/GLP-1R pathway (Jia et al., 2021). However, in hepatic fibrosis, ICA can exert an anti-autophagic effect by restoring mTOR expression (Algandaby et al., 2017). In physiologic states, autophagy is a widespread self-protective mechanism for maintaining cellular homeostasis. In pathological states, either over-inhibited or over-activated autophagy can promote the development of fibrosis. Some studies have confirmed that excessive or insufficient autophagy can promote the development of renal fibrosis (Shi et al., 2025; Chen et al., 2023). It is reasonable to speculate that the seemingly contradictory findings in fibrosis in different organs may imply that ICA has a bidirectional role in regulating the balance of autophagy. There is a lack of studies on the bidirectional regulation role for ICA and related metabolites. More basic research is necessary in the future to provide more theoretical support for this contradictory finding.

**FIGURE 3:**
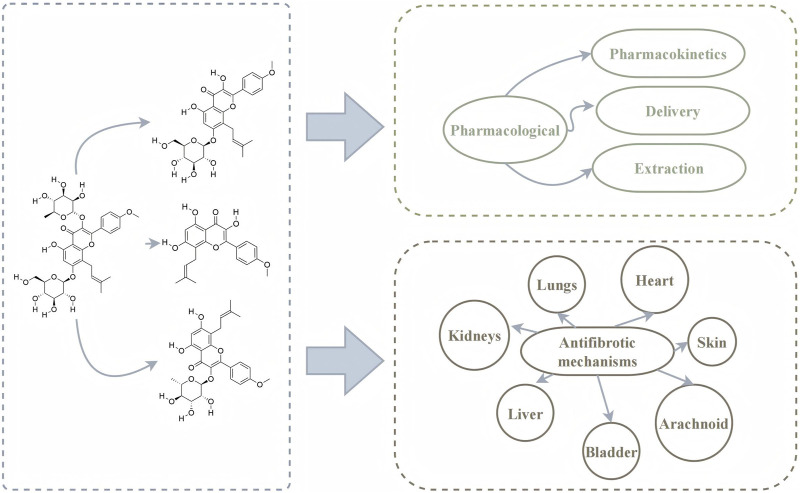
Graphical summary.

This review describes the pharmacological composition, pharmacokinetics, delivery, extraction and antifibrotic mechanisms of ICA and related metabolites (Figure 3). This review is helpful to promote the application of ICA and related metabolites in the future.

## Abbreviations

ICA, Icariin; TGF-β, transforming growth factor; TCM, Traditional Chinese medicine; NP, nanoparticle, F-ILD, Fibrosing interstitial lung diseases; TNF-α, tumor necrosis factor-α; IL-1β, interleukin-1β; α-SMA, Alpha smooth muscle actin, IL-4: interleukin-4, TLR: Toll-like receptor, DCM: Diabetic cardiomyopathy, NOS3, nitric oxide synthase 3; PDE5A, phosphodiesterase 5A; ROS, reactive oxygen species; CKD, Chronic kidney disease; EMT, epithelial-mesenchymal transition; SOD, superoxide dismutase; CAT, catalase; ECM, extracellular matrix; HSCs, hepatic stellate cells; GPX4, Glutathione peroxidase 4; MDA, malondialdehyde; CYP, Cyclophosphamide; CTGF, connective tissue growth factor; AMPK, AMP-activated protein kinase; NF-κB, nuclear factor kappa B.
